# Targeting the “undruggable” RAS - new strategies - new hope?

**DOI:** 10.20517/cdr.2019.21

**Published:** 2019-09-19

**Authors:** Britta Mörchen, Oleksandr Shkura, Raphael Stoll, Iris Helfrich

**Affiliations:** ^1^Skin Cancer Unit of the Dermatology Department, Medical Faculty, University Duisburg-Essen, West German Cancer Center, Essen 45147, Germany.; ^2^German Cancer Consortium (DKTK) partner site Düsseldorf/Essen, Essen 45147, Germany.; ^3^Biomolecular NMR, Faculty of Chemistry and Biochemistry, Ruhr University of Bochum, Bochum D-44780, Germany.; ^#^Both authors contribute equally.

**Keywords:** K-RAS, small molecules, immune checkpoints, PD-1, PD-L1

## Abstract

*K-RAS* is the most frequently mutated oncogene in solid tumors, such as pancreatic, colon or lung cancer. The GTPase K-RAS can either be in an active (GTP-loaded) or inactive (GDP-loaded) form. In its active form K-RAS forwards signals from growth factors, cytokines or hormones to the nucleus, regulating essential pathways, such as cell proliferation and differentiation. In turn, activating somatic mutations of this proto-oncogene deregulate the complex interplay between GAP (GTPase-activating) - and GEF (Guanine nucleotide exchange factor) - proteins, driving neoplastic transformation. Due to a rather shallow surface, K-RAS lacks proper binding pockets for small molecules, hindering drug development over the past thirty years. This review summarizes recent progress in the development of low molecular antagonists and further shows insights of a newly described interaction between mutant K-RAS signaling and PD-L1 induced immunosuppression, giving new hope for future treatments of *K-RAS* mutated cancer.

## Introduction

**Figure 1:**
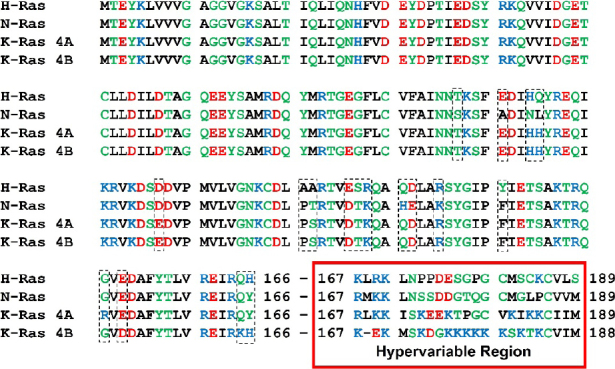
Amino acid sequences of RAS isoforms. The sequences of the RAS isoforms are compared and non-identical areas are marked by dashed rectangles. Amino acids 1-166 constitute the catalytic domain and amino acids 167-188/189 represent the hypervariable region. The amino acids are coloured based on their physico-chemical properties. Hydrophobic (black), polar/neutral (green), acidic (red) und basic (blue)

During evolution, cells have acquired the ability to respond to external stimuli through fine-tuned signaling cascades. To this extent, rat sarcoma (RAS)-proteins are small monomeric G-proteins that utilize various signal transduction pathways to regulate cell growth, differentiation, and apoptosis^[1]^. Due to their proto-oncogenic character, RAS proteins are a popular target in the field of cancer research^[2]^. The most prominent members of the RAS family are the isoforms N-RAS, H-RAS, K-RAS 4A and K-RAS 4B. These proteins emerge from proto-oncogenes, as their point-mutations are frequently present in different types of cancer^[3]^. The amino acid sequence of all four isoforms is shown in Figure 1. The first 166 amino acids constitute the catalytic G-domain and share a sequence identity of 89%. The subsequent region spanning residues 167 to 188 and 189, respectively, differ among the isoforms. Hence, this C-terminal domain of RAS proteins is denoted as the hypervariable region.

K-RAS 4A and 4B represent two splicing variants of the exon 4 of the K-RAS pre-mRNA and all four isoforms possess the C-terminal CaaX-Box. The cysteine at position 185 or 186 respectively is followed by two aliphatic amino acids and it is this cysteine that plays a crucial role in the post-translational processing of RAS. All RAS isoforms are farnesylated to enable their anchoring to the membrane. Additionally, K-RAS 4A can be palmitoylated at cysteine 180, N-RAS at cysteine 181, and H-RAS at cysteine 181 and 184. This additional palmitoylation increases the hydrophobicity of the protein. In contrast to this, K-RAS 4B is not palmitoylated but contains a poly lysine stretch (K175-K180) in the hypervariable region instead. This positively charged sequence facilitates a more transient anchorage to the negatively charged plasma membrane.

### RAS - function and structure

RAS proteins function as binary molecular switches in signal transduction. They exist in an active GTP-bound “ON”-state and an inactive GDP-bound “OFF”-state^[4]^. In the active state, the G-protein is able to bind to different effector proteins and transfer the signal. The intrinsic GTPase property enables the hydrolysis of GTP to GDP and hence the transition from the active to the inactive state. For the reactivation of RAS, the GDP nucleotide has to dissociate and the GTP nucleotide, which is present in a 10-fold excess, has to be incorporated. The intrinsic GDP-dissociation rate is 10^-5^ s^-1[5]^ and thus too low to be biologically significant. This rate is enhanced 10^4^-fold by guanine nucleotide exchange factors (GEFs)^[6]^. These GEFs are positive regulators of the RAS activity. Hitherto, the protein encoded by the cell division cycle gene 25 (Cdc25) and the son of sevenless (SOS) are the best understood GEFs for RAS. GTPase activating proteins (GAPs) function as counterparts to GEFs. They are essential for the hydrolysis of GTP as the inherent GTPase activity of RAS is only 10^-4^ s^-1^, which is amplified by GAPs by 10^3^-times.

**Figure 2:**
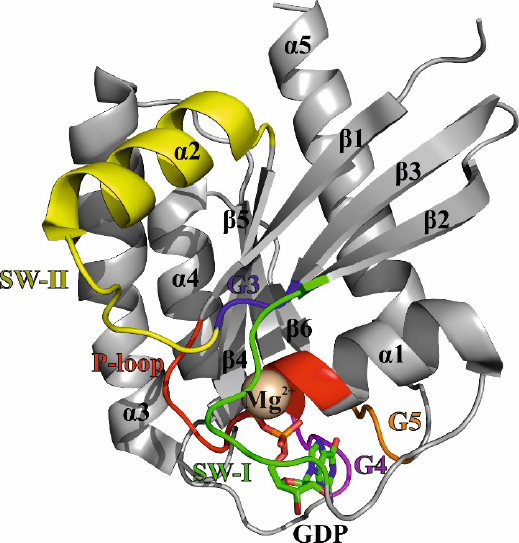
Crystal structure of K-RAS 4B GDP in ribbon representation (4EPY). The α-helices (α1-α5), β-sheets (β1-β6), the magnesium-ion (Mg^2+^), and the GDP nucleotide are labeled in black. The loops and switch regions are color-coded as follows: P-loop (red); switch I - SW-I (green); G3-loop (blue); switch II - SW-II (yellow); G4-loop (magenta); G5-loop (orange)

The three-dimensional structure of RAS proteins explains the molecular mechanism of their biological function as cellular protein switches [Figure 2]. The guanosine nucleotide binds to the G-protein conserved G-domain. The G-domain constitutes of six β-sheets and five surrounding α-helices which are connected with ten loops^[6]^.

Five of the ten loops that can be found between the secondary structural motifs are denoted as G1 to G5 motifs and participate in nucleotide binding. The G1-loop is glycine-rich region and utilizes the positive charge of lysine 16 to bind the β-phosphate of the nucleotide. The β-phosphate is additionally coordinated by serine 17 and a magnesium ion. The positive charge of magnesium is essential to stabilize the three negative phosphate groups of the nucleotide and, therefore, the G1-loop has also been described as the P-loop (GxxxGKS/T). The amino acids 32 to 38 form the G2-loop, also known as switch I. The threonine at position 35 is crucial in the binding of the γ-phosphate and the magnesium ion. In the presence of the γ-phosphate, the switch I region adopts the active conformation and participates in the effector binding^[4]^. The G3-loop includes the motif DxxG and shares glycine 60 with the neighboring switch II region, which comprises amino acids 60 to 76. The glycine 60 is the second amino acid to bind the γ-phosphate through a hydrogen bond. Like switch I, switch II changes its conformation upon binding of GTP and does not only take part in effector binding but also acts as an interaction site for GEFs and GAPs^[7]^. The binding of the guanosine nucleotide is established by the G4-loop (N/TKxD aa 116 to 119) as well as the G5-loop (SAK aa 145 to 147)^[8]^.

The conformational change of the protein from the inactive, GDP-bound state to the active, GTP-bound state has been coined as the loaded-spring mechanism^[8]^. In the active state, amino groups of threonine 35 (located in switch I) and glycine 60 (located in switch II) coordinate one oxygen atom of the γ-phosphate *via* hydrogen bonds. Upon hydrolysis of the γ-phosphate, the hydrogen bonds are broken and the flexible switch-loops spring back into their initial positions. Noteworthy, the loaded spring of the active state is not simply a rigid construct. The active state contains further two (sub-) states^[9]^. The difference between these two active states is the flexibility of the switches regions and the results in different affinities towards the effector proteins. In state 1, the switch regions exhibit a higher conformational fluctuation compared to state 2, which results in impaired binding to the effector RAF^[10]^. These conformational changes between the active and inactive conformation as well as the subdivision in active state 1 and 2 further emphasizes the dynamic nature of these switch regions of the RAS protein, an attribute referred to as polysterism^[11]^.

## K-RAS in cancer

### K-RAS signaling

K-RAS acts as a cellular switch to regulate essential intrinsic pathways^[12-14]^. Extracellular stimuli, e.g., binding of growth factors, hormones or cytokines, are leading to a dimerization of receptor tyrosine kinases, which in turn activate GEFs, such as SOS, which leads to a nucleotide exchange and an activation of K-RAS. In its wild-type (wt) state K-RAS forwards signals from growth factors, cytokines or hormones to the nucleus, thereby regulating crucial pathways such as proliferation, differentiation and cell growth^[15,16]^. GAPs are responsible to enhance the catalytic rate of K-RAS GTPase function, leading to the hydrolyzation of GTP, the replacement with GDP, resulting in the inactivation of K-RAS^[6,17]^. Activating somatic mutations of this proto-oncogene disrupt this complex interplay between GEFs and GAPs^[18,19]^. GAPs can no longer increase the GTP hydrolyzation of oncogenic K-RAS, leading to a constitutive active mutant (mut) form driving neoplastic transformations in many cancer entities^[20-22]^. With a frequency of 21.6% somatic point mutations in *K-RAS* are the most common mutations in the RAS gene of all human cancers, followed by an 8.0% incidence of *N-RAS* mutations and a 3.3% rate of *H-RAS* mutations^[23]^.

**Figure 3:**
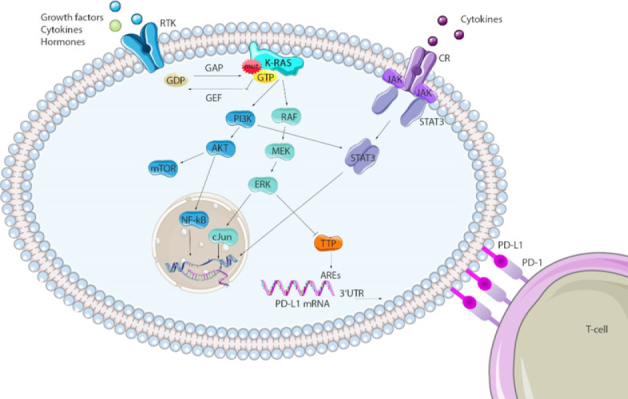
Major effector pathways of K-RAS and potential interactors. Mutations in *K-RAS* genes lead to constitutive GTP bound K-RAS proteins that activate downstream phosphorylation cascades. Major effector pathways dysregulated in *K-RAS* mut cancers are the MAPK (RAF/MEK/ERK) and the PI3K/AKT pathway. Transcription factors regulated by these pathways, as well as STAT3, can bind to the *PD-L1* gene, enhancing the transcription rate. TPP destabilizes PD-L1 mRNA at AREs on the 3’UTR region and can be inhibited via MEK signaling in *K-RAS* mut cancers, prolonging the PD-L1 mRNA’s half-life. This figure was partly created using SMART Servier Medical Art. ARE: AU-rich elements; CR: cytokine receptor; RTK: receptor tyrosine kinase; TTP: tristetraprolin

The mitogen-activated protein kinase (MAPK) signaling cascade is crucial for the regulation of cellular functions like differentiation and cell growth of normal cells. Constitutive activation of this pathway leads to uncontrolled cell proliferation, transformation, dissemination of cancer cells and is one of the major effector pathways deregulated in *K-RAS* mutant cancer^[24,25]^. The pathway consists of the kinases RAF, mitogen-activated protein kinase/extracellular receptor-stimulated kinase 1/2 (MEK), and extracellular receptor-stimulated kinase 1/2 (ERK), that are part of a phosphorylation cascade, downstream of activated GTP-bound RAS [Figure 3]. Once activated in the cytoplasm, ERK can activate proteins of the dynamic cytoskeletal complex that affect cell adhesion, trafficking, and movement^[26,27]^. In addition, it can also enter the nucleus and regulate various mitogenic transcription factors involved in stimulating cell proliferation^[28,29]^. The phosphoinositide 3-kinase (PI3K)/AKT pathway is the second major effector pathway downstream of mut *K-RAS* signaling. GTP-bound *K-RAS* can recruit and phosphorylate PI3K. Activated PI3K can phosphorylate the downstream serine/threonine kinase AKT, which in turn translocate from the plasma membrane to other cellular compartments, phosphorylating target proteins responsible for regulating cell growth, cell survival and entry of the cell cycle^[28,30]^. An important downstream target of AKT is the mammalian target of rapamycin (mTOR) complex 1, which results in lipid or nucleotide synthesis, as well as in protein translation^[31]^.

Nowadays, cancer therapy is able to inhibit both effector pathways downstream of *K-RAS* *via* selective MEK- (Binimetinib, Cobimetinib, Selumetinib, Trametinib)^[32-36]^, B-RAF- (Dabrafenib, Vemurafenib, Sorafenib, Encorafenib)^[32,37-40]^, or PI3K- (Idealisib, Buparlisib)^[41-44]^ inhibitors. However, continuous efforts over the past decades failed to develop novel therapies for *K-RAS* mutant cancer.

### *K-RAS* mutations as a key molecule driving chemoresistance

As there is no targeted therapy against *K-RAS* mut cancers available today, chemotherapy is still standard care in the treatment of cancer patients harboring mutations in this very oncogene. Pancreatic cancer, associated with a 90% chance of mutations in the *K-RAS* gene, is very poorly responsive to standard care first and second line gemcitabine/fluoropyrimidine chemotherapy due to metabolism-dependent drug resistances^[45,46]^. Metabolic reprogramming of *K-RAS* mut cancers is characterized by boosted glycolysis, glutaminolysis and pentose phosphate pathway, among others. Enhanced metabolism is not only providing more energy to the tumor, but has further impact on cell growth, rapid proliferation, invasion and drug resistance^[47]^. *K-RAS* and *MYC* oncogenes, as well as tumor suppressors like tumor protein p53, or phosphatase and tensin homolog (PTEN) are directly reprogramming a cancer cell’s metabolism in order to sustain unrestricted tumor growth, thereby creating a metabolism addiction^[47-49]^. The correlation between the strong addiction of cells to this oncogene and its impact on drug resistance highlights the big need to find specific *K-RAS* antagonists for more efficient future therapies.

## Very recent advances in developing low molecular weight antagonists of RAS

RAS proteins already were in the focus of medicinal chemical endeavors in the last millennium but unfortunately proved to be difficult targets. This previously earned RAS the inglorious title of being “undruggable”. During the past decade, tremendous progress has been made in modulating RAS by low molecular weight compounds, rendering RAS now indeed “druggable”. In principle, there are three common approaches to address RAS directly. These include to target the CaaX-Box, the nucleotide binding domain (NBD) or a shallow hydrophobic pocket including the switches I and II.

### The CaaX-Box of RAS as a conceptionally new target site

The first attempts to target the C-terminal CaaX-Box were based on the idea of interrupting the membrane binding and hence abolishing the signal transduction of RAS. The farnesyltransferase (FTase) is responsible for the farnesylation of all RAS isoforms at cysteine 185/186. The development of FTase inhibitors led to various products, of which some have entered clinical trials. Probably the most promising one is Tipifarnib that is currently in clinical trials^[50]^. However, studies have shown that Tipifarnib and other FTase inhibitors only affect the isoform H-RAS. The other isoforms, N-RAS as well as *K-RAS* 4A and 4B, are still prenylated by geranylgeranyl transferase^[51]^.

### The *G12C RAS* mutant as a special target

Targeting the NBD is difficult due to the high picomolar affinity of RAS towards the nucleotide GDP and GTP^[52]^, as well as the high micromolar abundance of intracellular GDP and GTP^[53]^. One opportunity to compete with these conditions unveils itself in form of the oncogenic mutant *K-RAS* *G12C*. The mutation of glycine 12 to cysteine establishes a nucleophilic target in proximity to the NBD for covalent binding of electrophilic inhibitors. SML-8-73-1 is a GDP-analogue which utilizes an electrophilic warhead to tether cysteine 12 and bind to the nucleotide binding pocket of *K-RAS*^[54]^. As a GDP-analogue it is able to lock *K-RAS* G12C in the inactive state. Another way to employ the solvent exposed cysteine anchor is based on the combination of an electrophile with a compound which is able to bind to the neighboring switch II region^[55,56]^. The crucial aspect of the interaction with the switch II is that the binding pocket is only present in the inactive GDP-bound state. In case of the covalent inhibitor 12 developed by Ostrem *et al*.^[56]^ the binding to switch II results in disorder of both switch regions and enhances the affinity of *K-RAS* G12C towards GDP, hence rendering the protein inactive and decreasing the affinity towards the effector RAF. As the covalent tethering only works for the G12C oncogene mutation but not for the other oncogene mutants such as *G12V*, *G12D* and *Q61H* the demand for allosteric based inhibitors binding to the RAS-surface becomes evident.

### Targeting the hydrophobic ligand binding site located next to the switch regions of RAS

**Figure 4:**
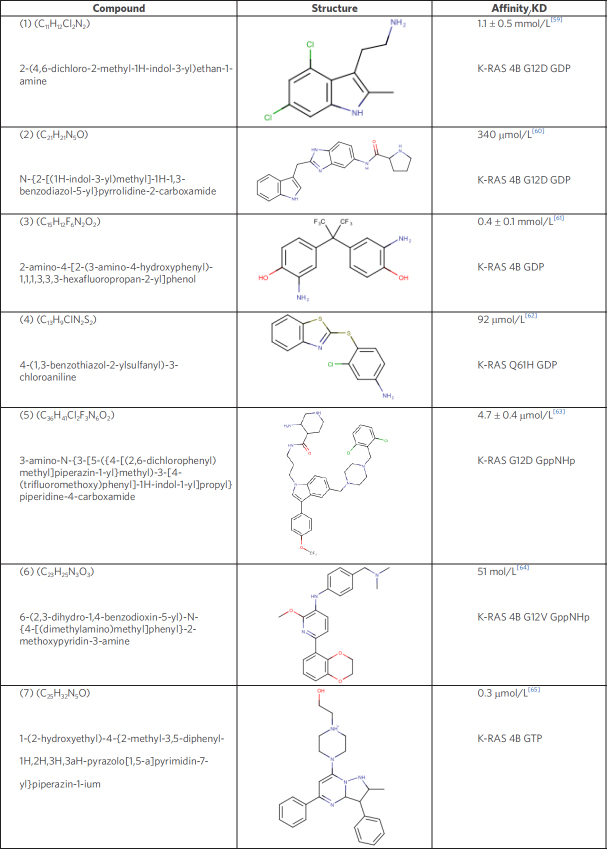
Low-molecular weight compounds targeting the RAS hydrophobic binding pocket

Pharmaceuticals aiming at the surface of RAS have been extensively studied and different compounds have been synthesized that target the GDP or GTP form. Various peptidomimetics^[57]^ and antibody fragments^[58]^ have been characterized as possible antagonists for RAS based tumors. This review however, will focus on low-molecular weight compounds for RAS. The ligands discussed in the following chapter are presented in Figure 4 and are numbered according to the order in this figure.

In 2012, two groups, one from Genentech and the other from Vanderbilt University, independently published the discovery of ligands that bind reversibly to a shallow hydrophobic pocket located next to switch I and II in the GDP-bound state of *K-RAS* 4B^[59,60]^. For both compounds, (1) and (2) the interaction site overlaps with the GEF binding site and hence interferes with the RAS-GEF complex formation. In addition, Schöpel *et al*.^[61,66]^ reported bisphenol derivatives to bind to the same hydrophobic binding pocket in the sub-millimolar to micromolar range. Bisphenol A (BPA) is widely used as a plasticizer best known for its endocrine disrupting character^[67]^. Notably, while BPA antagonizes the interaction between RAS and SOS, the fluorinated bisphenols BPAF and BPNH_2_ (3) even induce an allosteric activation of the GDP-bound *K-RAS* 4B. This induces a transition of RAS from the inactive, i.e., OFF-state, to a state in-between the OFF- and ON-state that is active^[61]^.

In 2017, Xie *et al*.^[62]^ reported on another ligand (4), that interacts with the hydrophobic binding pocket with the potential to hamper the binding of GTP. Further studies with non-small cell lung cancer (NSCLC) cell lines have shown that these compounds induce apoptosis in cell lines with oncogenic mutants of *K-RAS* 4B but not in wt *K-RAS* 4B cell lines.

Modulating the transition from inactive to active RAS is a reasonable strategy in disrupting the signal transition, but so is the interruption of the RAS-Effector complex of the active GTP-bound form. Molecular Dynamic simulations of the switch regions have identified a cluster of small binding domains which are present in all RAS isoforms^[63]^. Welsch *et al*.^[63]^ developed a so-called Pan-RAS inhibitor (5), which shows micromolar affinity *in vitro* for the RAS isoforms *K-RAS*, H-RAS, and N-RAS in the active state. This compound was tested in *in vivo* experiments using xenograft mouse tumor models and was shown to inhibit tumor growth over a period of 15 days of treatment. Notably, this study also reports certain levels of toxicity as well as off-target activity, which could hamper the indicated tumor growth inhibition.

As mentioned above, antibody fragment-based therapeutics have already been tested (Antibody 1998), but prove to be challenging regarding their permeability into the cell. Based on the structural information of the interaction between the antibody and its target site, Quevedo *et al*.^[64]^ have generated a low-molecular weight compound, Abd-7(6), which targets the same region with nanomolar affinity. Abd-7 binds to the switch regions and antagonizes the protein-protein interaction between RAS and its effectors. Abd-7 interacts with *K-RAS* G12V, which was bound to a non-hydrolysable GTP analogue, with an affinity of 51 nmol/L. However, its IC_50_ in cells is only 8 µmol/L that could suggest off-target effects and, once again, highlights the difference between molecular affinity *in vitro* and biological potency *in vivo*.

Finally, a novel inhibitor (7), by McCarthy *et al*.^[65]^ selectively interacts with both the GTP-bound wt and oncogenic mutants of *K-RAS* with sub-micromolar affinity but neither binds to the isoform H-RAS nor N-RAS^[65]^. The binding region of this pyrazolopyrimidine-inhibitor overlaps with the switch regions and thereby enables the inhibition of the RAS-RAF complex. The differentiation between the active and inactive state of RAS targets presents a promising opportunity for the future development of much needed selective inhibitors of activating oncogenic *RAS*-mutations, such as G12V, G12D, and Q61H.

**Figure 5:**
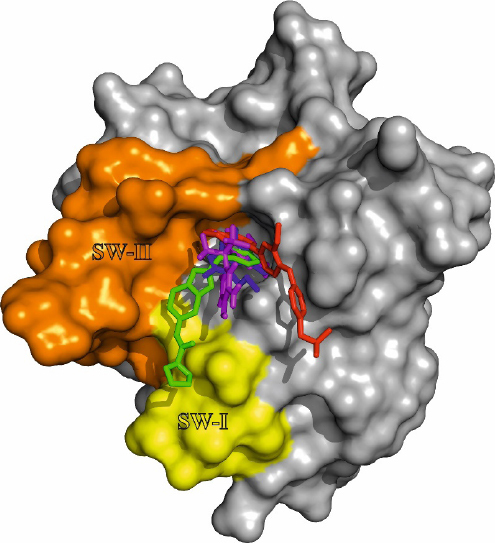
Ligand orientation in the hydrophobic binding pocket of K-RAS 4B. Co-crystal structure of (2) with K-RAS G12V (aa 1-169) [4EPY] in surface projection. The co-crystal structure of (1) [4DST] and (6) [6FA4] as well as (3) docked to [4DSO] have been aligned to 4EPY. The compounds are color-coded as follows: (1) blue; (2) green; (3) magenta; (6) red. The switch regions adjacent to the hydrophobic binding pocket are labeled switch I SW-I (yellow) and switch II SW-II (orange)

As described in the previous chapter, the presented ligands all bind to a hydrophobic binding pocket adjacent to the switch I and switch II region. To illustrate the binding orientation of the ligands the co-crystal structure of (2), [4EPY] was used in a surface projection. The co-crystal structures of (1) [4DST] and (6) [6FA4] as well as (3) NMR data-based docked onto 4DSO^[61]^ were then aligned to 4EPY [Figure 5].

## Influence of oncogenic *K-RAS* on cancer immunotherpy

### The programmed cell death protein 1 and its ligands

Evidence that inflammatory triggers play a critical role in the development and progression of cancer was already emerging at the beginning of the 20th century. Cancer patients were infected with bacteria to provoke inflammation which also affected the tumor cells^[68]^. For example, until today non-muscle invasive bladder cancer patients are receiving a therapy based on the infection of the bladder with *bacillus-calmette-guérin*^[69]^.

In 2018, the Nobel Prize for medicine and physiology was dedicated to the US American immunologist James Allison and the Japanese immunologist Tasku Honjo for their discovery of so called “immune checkpoints” and their therapeutic “mode of action” as negative immune regulators. Programmed cell death protein 1 (PD-1), also known as CD279, discovered in 1992 by Honjo *et al*.^[70]^ is a transmembrane immunoinhibitory receptor of the CD28 family, mostly expressed on active T-cells. Binding of PD-1 with its ligands (PD-L1/L2; known as CD274 and CD273) transmits a co-inhibitory signal in activated T-cells that promotes T-cell exhaustion, leading to tumor immune evasion^[71]^. However, contradictory results have suggested that PDL2 can serve as a negative and a positive regulator of T cell function. In 2004, Honjo *et al*.^[72]^ also discovered expression of PD-L1 on tumor cells, a potential strategy to suppress the host immune response and escape immune destruction. By using different preclinical models for melanoma and colon cancer he could show that a genetically or antibody-based PD-1 depletion leads to diminished hematogenous dissemination^[72]^. These experiments were the basis of the second immunotherapy approach of blocking PD-1/PD-L1 interactions. The anti-PD-1 antibody Nivolumab, in 2014 FDA approved for the treatment of advanced melanoma patients, is by the end of 2017and after more than 500 conducted clinical trials now approved for the treatment of nine different tumor entities including e.g., NSCLC, hodgkin lymphoma, squamous head and neck cancer or urothelial cancer^[73]^. Further FDA approved agents targeting the PD-1/PD-L1 axis e.g., Pembrolizumab (PD-1 inhibitor), Atezolizumab and Avelumab (both targeting PD-L1) are additionally as mono- or combination therapy in clinical use.

### Regulation of PD-L1 expression *via* oncogenic RAS signaling

Hanahan and Weinberg^[74]^ defined the capability of cancer cells to suppress the immune system, and to evade immune responses/destruction as a hallmark of cancer^[74]^. Moreover, the gain of mutations in cancer cells leads to changes in expression of surface molecules on the plasma membrane, so called neoantigens. The immune system is capable to recognize these neoantigens driving cytotoxic T-cells to destroy cancer cells. The immune system has certain breaks to dim or block this immune reaction, a mechanism preventing severe autoimmune responses, known as immune exhaustion^[75]^. As *K-RAS* mutant cancer is characterized by a high presentation of neoantigens that should activate T-cell response, these tumors have the potential to escape from immune destruction. Since therapeutic monoclonal antibodies targeting the immune checkpoints was a breakthrough by demonstrating long-term survival benefit for some patients with solid cancer, recent evidence suggest that there might be a connection between the regulation of the PD1/PD-L1 axis and *K-RAS* signaling.

Recent progress in developing small molecules against the formerly deemed “undruggable” *K-RAS* gives hope to patients suffering from *K-RAS* mutant cancer. However, there are still no clinically active drugs against mutant *K-RAS* variants available. Thus, understanding the complex interplay of oncogenic signaling and the immune system may help to circumvent mechanisms of resistance to immune checkpoint inhibitors. Preclinical and clinical data assumed that upregulation of PD-L1 on the cell surface of tumor cells seems to be a major driver of immune evasion. It was reported, that *K-RAS* mut NSCLC patients showed a prolonged overall survival after a Nivolumab treatment compared to *K-RAS* wt patients, who did not benefit from a PD-1 blockade^[76]^. Moreover, Garon *et al*.^[76]^ recently reported increased PD-L1 expression in *K-RAS* mut *vs*. *K-RAS* wt NSCLC tumors^[77]^. Therefore, it is of major importance to unravel the molecular mechanisms involved in the reciprocal interaction of activated *K-RAS* and presentation of PD-L1.

The best characterized stimulus from the tumor microenvironment that leads to up-regulation of PD-L1 expression is the inflammatory cytokine interferon (IFN) γ, acting *via* activation of the Janus kinase (JAK)/STAT pathway^[78]^. It has been shown that STAT3 binds to the PD-L1 promotor region, enhancing its transcription *in vitro* and *in vivo*^[79]^. This mechanism was proven in anaplastic large-cell lymphoma, T-cell lymphoma and in *K-RAS* mut NSCLC^[79-81]^. Recent evidence suggests that oncogenic RAS signaling can directly increase PD-L1 mRNA stability, which leads to enhanced PD-L1 surface expression and impaired tumor immune surveillance^[82]^. Whereas PD-L1 protein level were significantly increased in *BRAF* V600E mut melanoma cell lines resistant to BRAF inhibition, this effect was decreased both after using small interfering RNA (siRNA) mediated knockdown of ERK1/2, and after pharmacologic inhibition of MEK^[83,84]^. Jiang *et al*.^[83]^ further showed that depletion of c-Jun (transcription factor downstream of ERK) and STAT3 resulted in a synergistic decrease of PD-L1 surface expression, indicating a cooperation of RAS and STAT3 signaling in controlling PD-L1 expression in melanoma. MEK and ERK abrogation was further effective to minimize ectopic PD-L1 expression in *K-RAS* mut NSCLC cell lines^[81]^.

Moreover, the second major effector pathway of RAS, the PI3K/AKT pathway, also seems to play a role in regulating PD-L1 expression, as inhibition of either AKT or mTOR resulted in decreased PD-L1 levels in triple negative breast cancer (TNBC)^[85]^. This is in line with the observation that a knockdown of the PTEN (negative regulator of P13K) leads to elevated PD-L1 expression level in the same TNBC cell model, as well as in a murine lung squamous cell carcinoma model^[86]^. Inhibition of PI3K resulted in PD-L1 down-regulation in many tumor entities including *K-RAS* or *EGFR* mut NSCLC, renal cell carcinoma and melanoma^[83,87,88]^.

Coelho *et al*.^[82]^ were the first describing a novel translational control mechanism of PD-L1 expression *via* direct mut *K-RAS* signaling. By using a tamoxifen-inducible ER-KRAS^G12V^ fusion protein they could show a profound up-regulation of PD-L1 mRNA and surface protein level upon activation of oncogenic RAS. In contrast to IFN-γ dependent PD-L1 regulation, induction of PD-L1 *via* activated RAS is mediated by an increased half-life of the PD-L1 mRNA^[82]^. AU-rich elements (AREs) of 3’ untranslated regions (UTR) of the PD-L1 mRNA are targets of the ARE-binding protein tristetraprolin (TTP). Once TTP is physically bound to AREs, it destabilizes the PD-L1 mRNA. It was proven in lung cancer cell lines, that mut *K-RAS* driven MEK signaling reduces intracellular TTP levels, thereby prolonging PD-L1mRNA’s half-life and stability, which in turn increased the cells PD-L1 surface expression. Additionally, TTP restoration in *K-RAS* mut tumors resulted in enhanced anti-tumor immunity in murine colon cancer models^[82]^. Curiously, a TTP deficiency in macrophages and cytotoxic T-cells increases T-cell induced anti-tumor activities *via* modified cytokine expression^[89]^.

Interestingly, genomic alterations in the 3’UTR region of the *PD-L1* gene itself have an impact on the PD-L1 expression in some cancers^[90]^, which may indicate a shared TTP regulation mechanism of the PD-L1 mRNA, as it is the case in *K-RAS* mut cancers.

### Clinical implication of *K-RAS* status and PD-L1 expression in cancer

Continuous efforts in the past three decades failed to develop approved therapies targeting activated *K-RAS*. Although it is too early to claim clinical benefits, new therapeutic strategies can evolve from the knowledge about the interaction of these key molecules in tumor biology. So, the strategy to combine immunotherapies has brought new hope for *K-RAS*-dependent tumors. A more recent clinical study analyzed the impact of the individual *K-RAS* mutations on PD-L1 expression and clinical outcome in lung adenocarcinoma patients. They showed that exclusively G12V, and none of the other mutations in *K-RAS*, leads to a significant increase in PD-L1 expression^[91]^. This study further states a correlation between high PD-L1 expression and improved OS only in *K-RAS* mut cancer. Additionally, Falk *et al*.^[91]^ reported that high PD-L1 expressing immune cells in these patients correlated with poor OS, independent on the tumor’s *K-RAS* mutation. These findings show how heterogeneous *K-RAS* mutational status affects the immune system in lung adenocarcinomas, indicating a *K-RAS* dependent patient stratification for immunotherapy in future.

### Combination of small molecules and PD-1 inhibition in patients with *RAS* mutant tumors

Currently there are many preclinical analyses and clinical trials ongoing to target *K-RAS*-driven cancer. A broad range of novel therapeutic strategies, e.g., the usage of siRNA and exosome siRNA, monoclonal antibodies targeting the GTP-bound *K-RAS*, use of oligonucleotides to prevent membrane embedding and farnesyltransferase inhibitors implicate new hope. However, it will take some time before we can draw conclusions about the effectiveness of these new therapeutic approaches. Nevertheless, a currently recruiting phase 1b clinical trial, initiated by Targovax, is evaluating the effect of the multi-kinase inhibitor TG02 in combination with the anti-PD-1 antibody Pembrolizumab in patients with locally advanced primary tumors in *RAS* mutated colorectal cancers (ID: CT TG02-01). TG02, a second-generation RAS neo-antigen vaccine, selectively targets tyrosine kinase receptors (e.g., fms-like tyrosine kinase receptor-3), mitotic and transcriptional cyclin dependent kinases 1, 2, 3, 5, as well as lymphocyte-specific protein tyrosine kinase, proto-oncogene tyrosine-protein kinase Fyn, non-receptor tyrosine-protein kinase 2 and JAK 2^[92,93]^. TG02 is reported to induce apoptosis in cell lines and primary cells of acute myeloid leukemia, and lead to tumor regression in xenograft models^[93]^. In 2018, Su *et al*.^[94]^ reported induction of apoptosis, reduced tumor cell proliferation and a prolonged OS of mice harboring glioblastoma after TG02 treatment. Early exploratory clinical data indicate that TG02 induces immune response in patients with so called “hot tumors” which are rich in numbers of activated tumor-infiltrating T-cells compared to controls. Moreover, PD-1 expression was observed in both circulating and tumor-infiltrating T-cells. This further strengthens the rationale for combining TG02 with PD-1 checkpoint blockade. Whether this dual targeting would be advantageous compared with the previous therapeutic strategies still await clinical validation.

## Conclusion

*RAS* oncogenes are often activated by point mutations in a wide range of different cancer entities, thereby identifying RAS as an important clinical target. There are three RAS genes (*H-RAS, K-RAS* and *N-RAS*) whereas *K-RAS* is the most frequently mutated oncogene in cancer. Activating somatic mutations of this proto-oncogene occur in approximately 30% of human cancers driving neoplastic transformation. The *K-RAS* protein is lacking proper binding pockets for small molecules, resulting in no progress in drug development over the past thirty years. Recently new findings e.g., the identification of a new binding pocket interfering with the SOS-*K-RAS* interaction has aroused the interest to re-visit the “formerly undruggable” target. In addition, more recent insights into the interplay between mutant RAS and the PD-1/PD-L1 signaling provide a portfolio of synergistic treatment options through the additional use of inhibitors targeting the immune checkpoints. Nevertheless, although novel therapeutic directions are currently under investigation, the risk for induction of acquired resistance is also in clinically effective drugs expected given by the strong selective pressure applied to the genetically unstable cancer cells.

## Declarations

### Copyright

© The Author(s) 2019.
